# Use of Abdominal Binders after a Major Abdominal Surgery: A Randomized Controlled Trial

**DOI:** 10.7759/cureus.5832

**Published:** 2019-10-03

**Authors:** Summaya Saeed, Khaled Abdullah Rage, Amjad Siraj Memon, Sarah Kazi, Khursheed Ahmed Samo, Sana Shahid, Aun Ali

**Affiliations:** 1 Surgery, Civil Hospital, Karachi, PAK; 2 General Surgery, Civil Hospital, Karachi, PAK; 3 General Surgery, Dow University of Health Sciences, Karachi, PAK; 4 Obstetrics and Gynecology, Civil Hospital, Karachi, PAK; 5 Surgery, Fazaia Ruth Pfau Medical College (FRPMC), Karachi, PAK

**Keywords:** 6-minute walking test, visual analogue scale, american society of anesthesiologists classification, postoperative day, abdominal binder, abdominal surgery

## Abstract

Objective

To compare the effect of abdominal binder versus no binder after major abdominal surgery and cesarean section on various post-operative recovery parameters.

Materials and methods

This is a randomized controlled trial conducted at the Department of General Surgery and Obstetrics, Civil Hospital, Karachi, Pakistan. All those patients aged ≥18 years having abdominal surgery including elective and emergency abdominal surgery and cesarean sections with American Society of Anesthesiologists Class I-III were included in the study. Randomization was done using the sealed envelope method by the principal investigator. The intervention group wore an abdominal binder postoperatively while the control group did not use it. Mobilization and the pain status of both groups were evaluated on the first, fourth, and seventh days after surgery.

Results

Primary outcome variables were mobility, assessed via 6-minute walk test (6MWT) and postoperative pain, evaluated via visual analogue scale. There was no statistically significant difference in the 6MWT distance before (p = 0.278) and on postoperative day one of the surgery (p = 0.0762). However, the difference was significant on fourth (p < 0.001) and seventh day (p value < 0.001). With regards to the pain status, patients in the binder group reported significantly less postoperative pain on first, fourth, and seventh (p value < 0.001) day compared to the non-binder group.

Conclusion

The use of abdominal binder postoperatively significantly reduced pain and improved mobility in both obstetric and surgery patients.

## Introduction

Major abdominal surgeries primarily include surgeries involving gut manipulation in the form of resection, anastomosis or stoma formation. It is fairly common to see various complications following these surgeries that may require invasive treatment in the form of re-exploration or intensive care management [1]. These complications can be avoided altogether by enforcing rigorous peri- and postoperative care measures [2]. The use of abdominal binders postoperatively is one such measure that can greatly enhance the recovery process.

Abdominal binders are compression belts that encircle abdomen, commonly used to augment the recovery process after abdominal surgery like exploratory laparotomy, cesarean section, bariatric surgery, hysterectomy, or spinal surgery. Speed recovery not only promotes wound healing but also safeguards against deep vein thrombosis, hypostatic pneumonia, and muscle atrophy according to the latest enhanced recovery after surgery concept [3]. Both elastic and non-elastic binders are available in the market. Abdominal binder helps to reduce postoperative pain, distress, and hemorrhage after cesarean section [4]. These are also used in patients with spinal cord injury to support the abdomen, to maintain intraabdominal pressure, improve respiratory function and improve overall mobility [5]. They decrease postoperative pain, psychological stress, promote postoperative recovery and prevent abdominal wall dehiscence in open abdominal surgeries [6-8]. Abdominal binders are safe after midline laparotomy as these minimally affect respiratory mechanics, intraabdominal pressure and wound healing [9].

A number of clinical trials were conducted worldwide to assess the effect of wearing abdominal binders after open abdominal surgeries such as exploratory laparotomy, cesarean section, or ventral hernia repair. Various factors such as postoperative pain relief, lung function, coughing, psychological stress, intraabdominal pressure, seroma formation, mobility, wound dehiscence, and general well-being were studied. Bouvier et al. demonstrated that abdominal binders prevent abdominal wall dehiscence in 83% and lead to postoperative pain relief in 66% of patients [8]. Rothman et al. in a systematic review revealed improvement in physical function and psychological stress but non-significant improvement in postoperative pain relief [6]. Gustafsson et al. in a recent trial conducted on cesarean patients reported a significant reduction in postoperative pain following the use of abdominal binder (p = 0.019) [3].

Most of these studies were done in western countries and data from South Asia is lacking. The use of an abdominal binder following abdominal surgery is not very popular in local departments and hence its effect on various recovery parameters has not been wildly studied. This study will aim to study the effect of an abdominal binder on key postoperative recovery parameters such as mobility, postoperative pain and general well-being.

## Materials and methods

This randomized controlled trial was conducted at the Department of General Surgery, Civil Hospital, Karachi, Pakistan from July to December 2017 with the principal objective of comparing the effect of abdominal binder versus no binder on postoperative recovery parameters. The study protocol was approved by the institutional review board. Written informed consent was taken in each case prior to the start of the study. All those patients aged ≥18 years having abdominal surgery including elective and emergency abdominal surgery and cesarean sections with American Society of Anesthesiologists (ASA) Class I-III were included in the study. Patients with Basal Metabolic Index (BMI) ≥35, history of abdominal surgery one year back, stage IV malignancy, chronic obstructive pulmonary diseases or those undergoing laparoscopic surgeries were excluded from the study. Patients were also excluded if they had any orthopedic, neuromuscular, or circulatory disorder severe enough to preclude 6-minute walk test (6MWT) evaluation. The sample size was calculated using openEPI calculator taking mean pain scores in the binder group to be 8.7 ± 8.1 versus 13.3 ± 10.9 in the non-binder group, and came out to be at least 69 patients in each group.

Baseline parameters such as age, weight, height, BMI, ASA Class, surgical diagnosis and type of surgery were recorded on predesigned proforma. Randomization was done using the sealed envelope method by the principal investigator. The intervention group wore an abdominal binder postoperatively and the control group will not use it. Mobilization and the pain status of both groups were evaluated on the first, fourth, and seventh days after surgery. Mobilization was assessed via the 6MWT and postoperative pain was evaluated via visual analog scale (VAS). Data were analyzed using IBM Statistical Package for the Social Sciences (SPSS) Statistics for Windows, version 21.0. (IBM Corp., Armonk, NY, US). Mean and standard deviation was calculated for quantitative variables such as age, VAS pain score and 6MWT distance whereas frequencies and percentages were calculated for qualitative variables such as gender, type of surgery, and ASA class. An independent t-test was used to compare the means between the two groups taking p-value <0.05 as statistically significant.

## Results

A total of 150 patients consented to participate in the study out of which 10 were excluded on the basis of inclusion/exclusion criteria. The rest of 140 patients were randomized into two groups of 70 each according to the sealed envelope technique. There were 50 males and 90 females with an overall average age of 44 ± 20 years. The mean BMI was 25.7 ± 2.0 kg/m^2^. Details of baseline parameters according to each group are depicted in Table 1. The majority of the patients had ASA class II and III with only four patients belonging to ASA class I. The most common type of surgery performed was cesarean section in up to 50 of the patients followed by emergency and elective exploratory laparotomy (Table 2). There was no significant difference in duration of anesthesia (p = 0.674) and length of incision amongst the two groups (p = 0.736).

**Table 1:** Baseline characteristics of the participants

Baseline characteristics		Binder group	Non-binder group
Age		42 ± 21	45 ± 26
Gender	Males	28	22
	Females	42	48
Basal metabolic index (kg/m^2^)		25.5 ± 2.1	26.1 ± 1.7
Duration of anesthesia (hours)		3.2 ± 2.9	3.4 ± 2.7
Length of the incision (cm)		17.9 ± 7.1	18.3 ± 6.9
Length of stay (in days)		6 ± 5	7 ± 6

**Table 2:** Surgical characteristics of the participants

Surgical characteristics		Binder group (n)	Non-binder group (n)
American Society of Anesthesiologists class	I	1	3
	II	37	44
	III	32	23
Type of surgery	Cesarean section	23	27
	Emergency exploratory laparotomy	15	10
	Colectomy (Right, Left, Sigmoid)	4	6
	Low anterior resection	5	5
	Ventral hernia repair	3	2
	Elective laparotomies	8	12
	Roux-en-y gastrojejunostomy	1	2
	Whipple procedure	1	-
	Other surgery	7	9

Primary outcome variables were mobility assessed via 6MWT and postoperative pain evaluated via VAS pain score. There was no statistically significant difference in the 6MWT distance before (p = 0.278) and on first postoperative day of the surgery (p = 0.0762) (Table 3). However the difference was significant on fourth (p < 0.001) and seventh postoperative day (p < 0.001). With regards to the pain status, patients in the binder group reported significantly less postoperative pain on first, fourth, and seventh postoperative day (p < 0.001) compared to the non-binder group (Table 4). Nevertheless, there was no significant difference in the length of stay at the hospital amongst the two groups (p = 0.286).

**Table 3:** 6-minute walk test distance for binder (experimental) and non-binder (control) groups

6-minute walk test distance (m)		Preoperative mean	Postoperative day 1	Postoperative day 4	Postoperative day 7
Group	Binder	299 ± 33	120 ± 26	214 ± 40	242 ± 38
	Non-binder	308 ± 42	109 ± 30	162 ± 31	200 ± 50
p -Value		0.278	0.0762	<0.001	<0.001

**Table 4:** Visual analogue scale pain score for binder (experimental) and non-binder (control) groups

Visual analogue scale pain score		Postoperative day 1	Postoperative day 4	Postoperative day 7
Group	Binder	3.64 ± 1.92	1.52 ± 2.03	0.86 ± 1.81
	Non-binder	6.08 ± 1.78	4.11 ± 2.60	2.99 ± 2.01
p-Value		<0.001	<0.001	<0.001

## Discussion

Major abdominal surgery is a source of huge stress for the body. The body has its own ways of coping with the stress of surgery, however the postoperative period needs to be optimized to augment the recovery process. Morbidity associated with such surgeries includes wound-related complications and pain at the incision site. It has been recognized that abdominal binders can fasten the recovery process and promote wound healing [10]. Compression increases blood flow and reduces swelling, both of which are key components of the healing process. This study was conducted to study the effect of wearing a binder on postoperative recovery.

Primary outcome variables for the study were mobility and postoperative pain. Mobility was checked by 6MWT and pain assessed via VAS. The 6MWT was chosen because of its excellent test-retest reliability and performance in monitoring surgical outcomes [11-14]. Whereas the VAS pain score is a widely used tool to assess the degree of pain in clinical research [15].

The present study reported significantly lower postoperative pain in the binder group as assessed via the VAS score. This was consistent with the findings of Arici et al. who also demonstrated significantly lower pain scores in the binder group [16]. Our study population included women undergoing cesarean section. Many past studies investigating the role of abdominal binder in reducing postoperative pain after cesarean section reported lower pain scores [3,4]. Gillier et al., however, reported no significant difference amongst VAS pain score between the binder and non-binder group [17]. Stoker et al. in a recent integrative review concluded that use of abdominal binder postoperatively provides pain relief, improves patient satisfaction, and reduces psychological distress [18]. Rothman et al. did report decreased psychological distress, however, their effect on postoperative pain was unclear [6].

The second key parameter assessed in the study was the patient’s postoperative mobility status. To begin with, the preoperative mean distance was not significantly different amongst the two groups (p = 0.278). Binder group showed significantly better mobility on the fourth and seventh postoperative day but not on day one. This was consistent with the findings of Cheifetz et al. and Arici et al. [9,16]. This can partly be explained by the assumption that patients are hesitant to move after major surgery fearing that their stitches might break loose, however, using abdominal binder gives them a sense of added assurity regarding the wound. Although wearing binder significantly reduced postoperative pain and improved physical function, there was no significant difference in the length of stay amongst the two groups.

## Conclusions

Complications secondary to surgeries can be avoided by adequate peri- and postoperative care. Abdominal binder helps to reduce postoperative pain, psychological stress, promote postoperative recovery and prevent abdominal wall dehiscence in open abdominal surgeries. This study was conducted to determine the effect of an abdominal binder on key postoperative recovery parameters. When compared with the control group, the use of an abdominal binder after surgery, significantly improved mobility and reduced pain in both obstetric and surgery patients.
